# Platelet factor XIII-A regulates platelet function and promotes clot retraction and stability

**DOI:** 10.1016/j.rpth.2023.100200

**Published:** 2023-06-07

**Authors:** Joanne L. Mitchell, Gemma Little, Alexander P. Bye, Renato S. Gaspar, Amanda J. Unsworth, Neline Kriek, Tanya Sage, Alexander Stainer, Ibidayo Sangowawa, Gael B. Morrow, Ricardo N. Bastos, Susan Shapiro, Michael J.R. Desborough, Nicola Curry, Jonathan M. Gibbins, Claire S. Whyte, Nicola J. Mutch, Christopher I. Jones

**Affiliations:** 1 Institute for Cardiovascular Research, University of Birmingham, Birmingham, UK; 2 Institute for Cardiovascular and Metabolic Research, School of Biological Sciences, University of Reading, Reading, UK; 3 School of Pharmacy, University of Reading, Reading, UK; 4 Heart Institute, University of Sao Paulo School of Medicine, Sao Paulo, Brazil; 5 Department of Life Sciences, Faculty of Science and Engineering, Manchester Metropolitan University, Manchester, UK; 6 Oxford University Hospitals NHS Foundation Trust, Blood Theme Oxford Biomedical Research Centre, Oxford, UK; 7 Radcliffe Department of Medicine, University of Oxford, Oxford, UK; 8 Oxford Nanoimaging, Oxford, UK; 9 Aberdeen Cardiovascular & Diabetes Centre, School of Medicine, Medical Sciences and Nutrition, Institute of Medical Sciences, University of Aberdeen, Aberdeen, UK

**Keywords:** clot retraction, factor XIII, fibrinogen, fibrinolysis, platelet activation, platelet

## Abstract

**Background:**

Factor XIII (FXIII) is an important proenzyme in the hemostatic system. The plasma-derived enzyme activated FXIII cross-links fibrin fibers within thrombi to increase their mechanical strength and cross-links fibrin to fibrinolytic inhibitors, specifically α_2_-antiplasmin, to increase resistance to fibrinolysis. We have previously shown that cellular FXIII (factor XIII-A [FXIII-A]), which is abundant in the platelet cytoplasm, is externalized onto the activated membrane and cross-links extracellular substrates. The contribution of cellular FXIII-A to platelet activation and platelet function has not been extensively studied.

**Objectives:**

This study aims to identify the role of platelet FXIII-A in platelet function.

**Methods:**

We used normal healthy platelets with a cell permeable FXIII inhibitor and platelets from FXIII-deficient patients as a FXIII-free platelet model in a range of platelet function and clotting tests.

**Results:**

Our data demonstrate that platelet FXIII-A enhances fibrinogen binding to the platelet surface upon agonist stimulation and improves the binding of platelets to fibrinogen and aggregation under flow in a whole-blood thrombus formation assay. In the absence of FXIII-A, platelets show reduced sensitivity to agonist stimulation, including decreased P-selectin exposure and fibrinogen binding. We show that FXIII-A is involved in platelet spreading where a lack of FXIII-A reduces the ability of platelets to fully spread on fibrinogen and collagen. Our data demonstrate that platelet FXIII-A is important for clot retraction where clots formed in its absence retracted to a lesser extent.

**Conclusion:**

Overall, this study shows that platelet FXIII-A functions during thrombus formation by aiding platelet activation and thrombus retraction in addition to its antifibrinolytic roles.

## Essentials

• Platelet factor XIII-A (FXIII-A) is present in abundance inside platelets.
• This study examines and characterises the roles of FXIII-A in platelet activation.
• Platelet FXIII-A regulates platelet activation and platelet sensitivity to stimulation.
• Platelet FXIII-A is involved in regulating blood clot compaction and clot stability.

## 1 Introduction

Platelets are integral to the formation and stabilization of the hemostatic plug following injury. During clot formation, platelets facilitate the tightening of the fibrin network to form dense clots that are effective in plugging the wound site without blocking the damaged vessel [[1], [2], [3]]. Platelet-rich areas of clots are extremely compacted, with platelet-associated fibrin being resistant to lysis [4].

Factor XIII (FXIII) is a transglutaminase proenzyme that, when activated, can form cross-linked amide bonds in protein substrates. There are 2 separate pools of FXIII; the plasma FXIII-A_2_B_2_ with 2 catalytic A subunits and 2 inhibitory carrier B subunits [5] and the cellular pool, composed of a homodimer of the A subunit (FXIII-A) [6,7]. The role of plasma FXIII is well defined, as it cross-links fibrin fibers to increase the mechanical stability of thrombi [8,9] and cross-links fibrinolytic inhibitors to fibrin [[9], [10], [11]] to increase fibrinolytic resistance. Cellular FXIII-A is found in numerous cell types including platelets [[12], [13], [14]], which contain an abundance of FXIII-A within their cytoplasm [6,7]. Cellular FXIII-A can be activated nonproteolytically by an intracellular influx of calcium [15,16] to FXIII^o,low^, which has different substrate affinity to its proteolytically cleaved counterpart [16]. We have previously showed that FXIII-A is translocated from the cytoplasm to the external membrane upon platelet activation [17]. Upon its externalization on activated platelets, platelet FXIII-A has the capacity to function in extracellular cross-linking reactions and stabilizes thrombi alongside activated plasma FXIII (FXIIIa) by cross-linking α_2_-antiplasmin to fibrin [17].

The high abundance of FXIII-A in platelets suggests that it could be involved in platelet function. It is currently unknown if platelet FXIII-A is involved in aspects of platelet biology, such as platelet activation and shape change. FXIII-A has many intracellular substrates including cytoskeletal proteins, such as actin, myosin, filamin, and vinculin [[18], [19], [20]], suggesting that it may be involved in cytoskeleton-mediated processes. The platelet cytoskeleton facilitates shape change [21] and degranulation [22] and drives clot retraction via the α_IIb_β_3_ receptor [23], all of which contribute to thrombus formation, stabilization, and lysis. Platelets can also undergo a morphologic change to form a “procoagulant” phenotype where phosphatidylserine is exposed on the platelet surface [24]. Previous studies have indicated that platelet FXIII-A may function in regulating thrombus size by altering α_IIb_β_3_ adhesiveness toward fibrinogen and converting platelets from adherent to procoagulant [25,26]. In this study, we determined the contribution of platelet FXIII-A in platelet activation, shape change, and spreading and defined the contribution of FXIII-A to clot retraction.

## 2 Methods

### 2.1 Patients

Peripheral blood was collected into vacutainers containing 3.2% sodium citrate (Greiner Bio-one) on 2 separate occasions from patients with FXIII deficiency (*n* = 2) with informed consent and ethical approval obtained from the Oxford Biobank (ORB Research Tissue Bank ethics approval 09/H0606/5+5).

Patient 1 was a female of Polish ethnicity aged 45 years on appointment (appt) 1 and 46 years on appt 2. Last FXIII treatment was given 25 days prior to appt 1 and 21 days prior to appt 2. FXIII was given in the form of 1250 units of fibrogammin. Phenotype was severe FXIII deficiency with multiple bleeding episodes, which was diagnosed in childhood. Baseline FXIII level was 2% (0.02 IU/mL). Platelet count (250 × 10^9^/L) and mean platelet volume (9.6 fL) were within the normal ranges.

Patient 2 was a male of Pakistani ethnicity aged 21 years on appt 1 and 22 years on appt 2 who was not related to patient 1. Last FXIII treatment was given 22 days prior to appt 1 and 19 days prior to appt 2. FXIII was given in the form of 2000 units of fibrogammin. Phenotype was severe FXIII deficiency with multiple bleeding episodes, which was diagnosed in infancy. Baseline FXIII level was 4 % (0.04 IU/mL). Platelet count (244 × 10^9^/L) and mean platelet volume (9.6 fL) were within the normal ranges.

### 2.2 Plasma and platelet preparation

Blood was taken from normal healthy male and female individuals aged >18 years who were not taking aspirin or anti-inflammatory medication, with informed consent and approval obtained from the University of Reading Research Ethics Committee (UREC 20/20). Platelet counts and mean platelet volume were within normal ranges for healthy donors. Peripheral blood was taken into 3.2% sodium citrate vacutainers, and the first 3 mL from each blood draw was discarded. Platelet-rich plasma (PRP) was prepared by centrifuging whole blood at 102 × *g* for 20 minutes at room temperature (RT). Platelets were isolated from PRP by centrifuging for 10 minutes at 1413 × *g* with the addition of 1:3 volume/volume (v/v) acid citrate dextrose (ACD; 85 mM sodium citrate, 71 mM citric acid, and 100 mM glucose) and 1.25 μg prostaglandin I2. The platelet pellet was washed once with modified Tyrode’s N-2-hydroxyethylpiperazine-N-2-ethane sulfonic acid buffer (Tyrode’s; 134 mM NaCl, 2.9 mM KCl, 0.34 mM Na_2_HPO_4_, 20 mM hydroxyethylpiperazine-N-2-ethane sulfonic acid, 1 mM MgCl_2_, and 5 mM glucose; pH 7.4) with an additional 1.25 μg of prostaglandin I2 and 1:3 v/v ACD before 10 minutes of centrifugation at 1413 × *g*. Washed platelets were resuspended in Tyrode’s at a concentration of 4 × 10^8^/mL and then rested for 30 minutes at 30 °C before use. Plaelet cytoplasm was isolated from washed platelet preparations per the Supplementary Methods.

### 2.3 Clot retraction

Clot retraction was performed on reconstituted PRP made from washed platelets from normal donors or FXIII-deficient patients (4 × 10^8^/mL final) and either pooled normal plasma or FXIII-deficient plasma (Affinity Biologicals). We used a transglutaminase inhibitor (1,3-dimethyl-2-[(2-oxopropyl) thio]imidazolium chloride; TGI; Zedira) that is capable of crossing the plasma membrane in resting platelets to inhibit FXIII-A. TGI can be detected in the platelet cytoplasm by mass spectrometry, (Supplementary Figure), detailed mass spectrometry methods and results are in the Supplementary Information. PRP was incubated in round-bottomed nonsiliconized glass tubes with or without TGI (1 mM) for 30 minutes at 37 °C. Activation mix containing thrombin (1 U/mL) and CaCl_2_ (2 mM) was added to initiate clotting around a glass pipette. Clotting proceeded for 30 minutes at 37 °C before clots were photographed and weighed.

### 2.4 Flow cytometry

#### 2.4.1 Measurement of platelet fibrinogen binding to detect integrin α_IIb_β_3_ activation

Washed platelets (4 × 10^7^/mL) from normal donors and FXIII-deficient patients were stimulated with or without TGI with 9,11-dideoxy-11α,9α-epoxymethanoprostaglandin F2α (U46619; 1 μM, Sigma-Aldrich), thrombin receptor activator peptide 6 (TRAP-6; 5 μM, Bachem), cross-linked collagen-related peptide (CRP-XL; 1 μg/mL), or adenosine diphosphate (ADP; 5 μM) for 20 minutes at 37 °C in the presence of fluorescein isothiocyanate (FITC)–labeled rabbit anti-fibrinogen antibody (1 mg/mL, Dako), which only measures platelet-released fibrinogen as no exogenous or plasma fibrinogen is present in the assay. Samples were fixed with 0.2% formyl saline (FS) and analyzed using a BD Accuri C6 Plus Flow Cytometer (BD). Platelets were gated using forward scatter and side scatter profiles, and data were collected from 5000 events within the platelet gate per sample and analyzed using inbuilt BD Accuri C6 plus software.

#### 2.4.2 Procoagulant platelet formation

Washed platelets (4 × 10^8^/mL) from normal controls were preincubated at 37 °C for 30 minutes with or without TGI before stimulating with dual agonists CRP-XL (1 mg/mL) and thrombin (1 U/mL) in the presence of CaCl_2_ (2 mM) and APC-labeled Annexin V (Life Technologies) for 15 to 60 minutes. The agonist mix was diluted in Tyrode’s buffer and analyzed using a BD Accuri C6 Plus Flow Cytometer collecting a minimum of 5000 events.

#### 2.4.3 Actin polymerization analysis

Actin polymerization in platelets was measured by flow cytometry as previously described [27]. Washed platelets (4 × 10^8^/mL) from normal controls were preincubated at 37 °C for 30 minutes with or without TGI (1 mM) before leaving unstimulated or stimulating for 20 minutes at 37 °C with CRP-XL (10 mg/mL) or thrombin (1 U/mL). Samples were fixed with 0.2% FS and permeabilized with 0.1% Triton X-100. Platelets were centrifuged at 1000 × *g* for 10 minutes at 4 °C and resuspended in Tyrode’s containing FITC-labeled phalloidin for 1 hour at RT. Platelet phalloidin content was analyzed using a BD Accuri C6 Plus Flow Cytometer by collecting 10,000 events per sample.

#### 2.4.4 PPAnalysis

Plate-based flow cytometry to measure platelet activation over a range of agonist concentrations was performed as described [28]. Briefly, fibrinogen binding (FITC antifibrinogen) and P-selectin exposure (PE-Cy5-anti-CD62P, BD) were measured in PRP after stimulation for 20 minutes at RT to ADP (0-30 μM), CRP-XL (0-3 μg/mL), or TRAP-6 (0-15 μM). Fibrinogen measured in this assay was from both platelet and plasma sources, and no additional exogenous fibrinogen was added to PRP. Flow cytometry was carried out by detecting 5000 events and analyzed using a BD Accuri C6 Plus Flow Cytometer.

### 2.5 Confocal microscopy

#### 2.5.1 Platelet spreading

Glass coverslips or Ibidi μ-chambers were coated with either 100 μg/mL collagen, 100 μg/mL collagen, and 1 U/mL thrombin or 100 μg/mL fibrinogen; washed with phosphate buffered saline (PBS); and blocked with 5-mg/mL bovine serum albumin (BSA). Washed platelets (2 × 10^7^ /mL) were allowed to adhere and spread for 60 minutes at 37 °C; in some cases, a fluorescent cross-linking probe for FXIII-A activity was incorporated—N-(tetramethylrhodaminyl)cadaverine (N-TAMRA-cadaverine; Zedira, ex 547, em 573). Nonadhered platelets were removed before fixing with 0.2% FS and permeabilized with 0.1% Triton-X100. Platelets were stained for F-actin using either Alexa-647 or Alexa-488 phalloidin (1:500). Coverslips were mounted on glass slides using Gold antifade mounting media and imaged using either a Zeiss LSM710 confocal microscope with 63 × 1.40 oil immersion objective and Zen 2012 software or a Nikon Ti-confocal system with 100× oil immersion objective and NIS-Elements image acquisition 3.1 software. Three randomly selected fields of view were captured for image analysis. Images were analyzed using ImageJ (National Institutes of Health) to determine the number of cells adhered and surface area coverage.

#### 2.5.2 Immunocytochemistry

Washed platelets (2 × 10^8^/mL) were either resting or activated with TRAP-6 (10 μM) in the presence of 4-μg/mL eptifibatide (Sigma-Aldrich) under stirring conditions for 3 minutes at 37 °C before fixation with 4% final v/v paraformaldehyde for 15 minutes. Platelets were pelleted at 950 × *g* for 10 minutes and then resuspended in 15% (v/v) ACD in PBS twice. Platelets were finally resuspended in 1% (weight/volume) protease-free BSA in PBS before adhering to poly-L-lysine–coated glass coverslips at 37 °C for 90 minutes. Coverslips were washed with PBS and blocked with BSA before staining. FITC-conjugated anti-FXIII-A (Zedira) was diluted to 1 μg/mL in 1% BSA, 0.2% Triton X-100, and 2% donkey serum and incubated at 4 °C overnight. Samples were washed with PBS before and after fixation with 4% paraformaldehyde and mounted on slides using Gold Antifade mounting media. Samples were imaged using Nikon Ti-confocal system with 100× oil objective and NIS-Elements image acquisition 3.1 software and analyzed using ImageJ.

#### 2.5.3 Thrombus formation under flow

Thrombus formation under flow was measured as described previously [29] in whole blood from normal healthy individuals and FXIII-deficient patients. Cellix Vena8 Fluoro+ biochips were precoated with collagen (100 μg/mL) or fibrinogen (100 μg/mL). Whole citrated blood was labeled with 3,3’-dihexyloxacarbocyanine iodide (DiOC6; 4 μg/mL) and treated for 30 minutes prior to perfusion with either TGI (1 mM) or vehicle control (0.01% dimethylsulphoxide). Blood was perfused for 10 minutes to form platelet thrombi at shear rates of 500/s or 1000/s. In some cases, CaCl_2_ (5.5 mM) and tissue-type plasminogen activator (20 nM; Sigma-Aldrich) were included with AF647-labeled fibrinogen (75 μg/mL; Life technologies) to form platelet-fibrin thrombi. Thrombi were allowed to form fully before switching flow to Tyrode’s containing tissue-type plasminogen activator (125 nM), and they were lysed to completion. Single z-slice images were taken every 2 to 4 seconds using a Nikon A1R fluorescence confocal microscope at 20× magnification. Data were analyzed using Image J by measuring fluorescence intensity of DiOC6 and AF647-fibrin(ogen) over time, which corresponded to thrombus size.

#### 2.5.4 Stochastic optical reconstruction microscopy

Ibidi μ-Slide 8 well glass bottom slides were coated with either 100 μg/mL collagen or 100 μg/mL fibrinogen, washed with PBS, and blocked with 5 mg/mL BSA. Platelets (2 × 10^7^) were allowed to adhere and spread for 60 minutes at 37 °C before fixing with 0.2% FS and permeabilizing with 0.1% Triton X-100. Platelets were stained for F-actin using stochastic optical reconstruction microscopy (STORM)–appropriate antibodies Alexa-532–labeled phalloidin (1:500) and unlabeled primary polyclonal rabbit anti–FXIII-A (Zedira) with Alexa-647–labeled goat anti-rabbit secondary. Platelets were submerged in B-cubed buffer (Oxford Nanoimaging Ltd [ONI]), and direct STORM images were acquired using a Nanoimager S Mark IB from ONI with 561-nm and 640-nm lasers. To increase resolution, imaging was performed in total internal reflection flourescence mode.

#### 2.5.5 Data analysis

Data were analyzed in GraphPad Prism 8.3.0. Image analysis was performed in FIJI by ImageJ. The STORM data were processed via NimOS from ONI. Statistical analysis was performed using Students *t*-test for 2 sample comparisons on all studies comparing inhibitor treatment to vehicle control. Analysis of variance was used for multiple comparisons. *P* < .05 was considered significant. Statistical analyses were not performed on studies comparing patients vs controls due to the small patient sample size.

## 3 Results

### 3.1 Platelet FXIII-A is present in abundance in healthy platelets and partially colocalizes with actin and actin nodules

**Figure 1:**
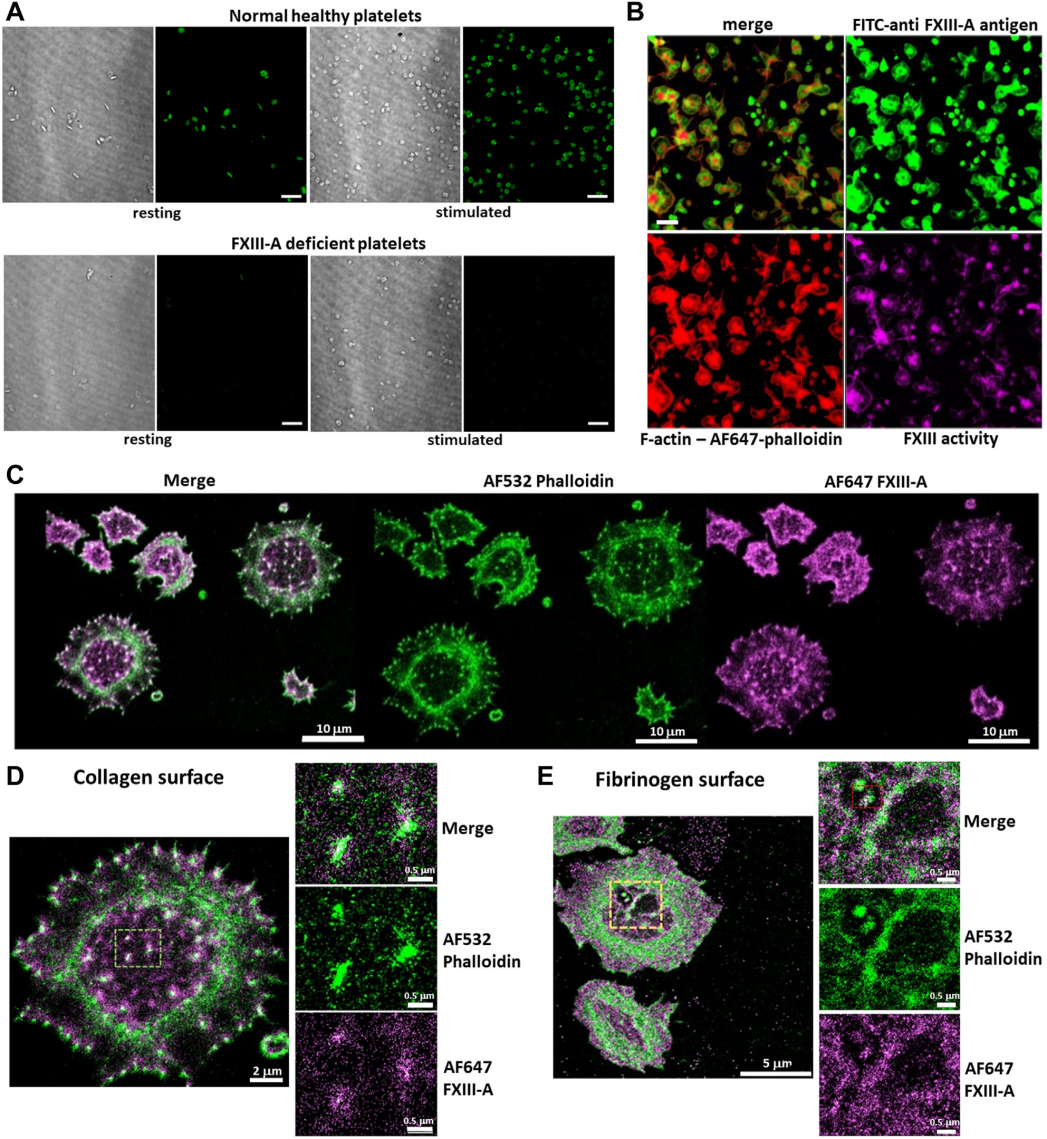
Platelet factor FXIII-A (FXIII-A) is abundant in healthy platelets and partially colocalizes with actin and actin nodules. Platelets were isolated from whole blood from normal healthy donors and factor XIII (FXIII)–deficient patients. (A) Immunocytochemistry was performed using fluorescein isothiocyanate (FITC) anti–FXIII-A antibody to detect platelet FXIII-A content in fixed, permeabilized platelets that were resting or stimulated with 10-μM thrombin receptor activator peptide 6 (TRAP-6) for 3 minutes. Normal healthy platelets (top panel), and FXIII-A–deficient platelets (bottom panel). Images representative of normal controls (*n* = 3) and FXIII-deficient patients (*n* = 2). Scale bar represents 20 μm. (B) Normal healthy platelets were spread on collagen (100 μg/mL) and thrombin (1 U/mL)–coated slides in the presence of a fluorescent probe, N-TAMRA cadaverine (ex 547), to measure FXIII-A activity for 45 minutes. Cells were then fixed, permeabilized, and stained using AF647-phalloidin to detect actin and FXIII-A using FITC anti–FXIII-A antibody. Scale bar represents 5 μm. (C–E) Normal platelets were washed and spread at 37 °C for 60 minutes on either collagen (100 μg/mL) or fibrinogen (100 μg/mL). Platelets were fixed, permeabilized, and stained with stochastic optical reconstruction microscopy (STORM)–appropriate AF532-labeled phalloidin anti–FXIII-A primary antibody with STORM-appropriate AF647-labeled secondary. Platelets were submerged in ONI Imaging Buffer for direct STORM imaging and imaged in Total internal refractive flourescence (TIRF). (C) Representative images of platelets spread on collagen: merged image (left), AF532-phalloidin (middle; green), and AF647-FXIII-A (right; magenta). Scale bar represents 20 μm. (D) Close-up image of a single platelet spread on collagen (left), with close-up images (right) of actin nodules in merged image (top), AF532-phalloidin (middle; green), and AF-FXIII-A (bottom; magenta). Scale bar on left represents 2 μm, and scale bars on right represents 0.5 μm (E) Close-up image of a single platelet spread on fibrinogen (left), with close-up images (right) of actin nodules in merged image (top), AF532-phalloidin (middle; green), and AF-FXIII-A (right, bottom; magenta). Scale bar on left represents 2 μm, and scale bars on right represent 0.5 μm.

Immunocytochemistry confirmed that platelets contained an abundance of FXIII-A antigen and that no traceable FXIII-A antigen was found in FXIII-deficient platelets (Figure 1A). Intracellular FXIII-A cross-linking activity was visualized in normal stimulated platelets using N-TAMRA-cadaverine, a fluorescent activity probe that acts as an amine donor and fluoresces when it is cross-linked into substrates by the transglutaminase activity of activated FXIII (Figure 1B). To determine the location of FXIII-A within platelets, we performed direct STORM imaging. FXIII-A was present in abundance throughout spread platelets and partially colocalized with the actin cytoskeleton (Figure 1C). FXIII-A was also identified in actin nodules in platelets spread on both collagen (Figure 1D) and fibrinogen (Figure 1E). These data suggest that a pool of FXIII-A is retained upon platelet activation in addition to the FXIII-A exposed on the platelet surface [17].

### 3.2 Inhibition or absence of platelet FXIII-A reduces platelet spreading

**Figure 2:**
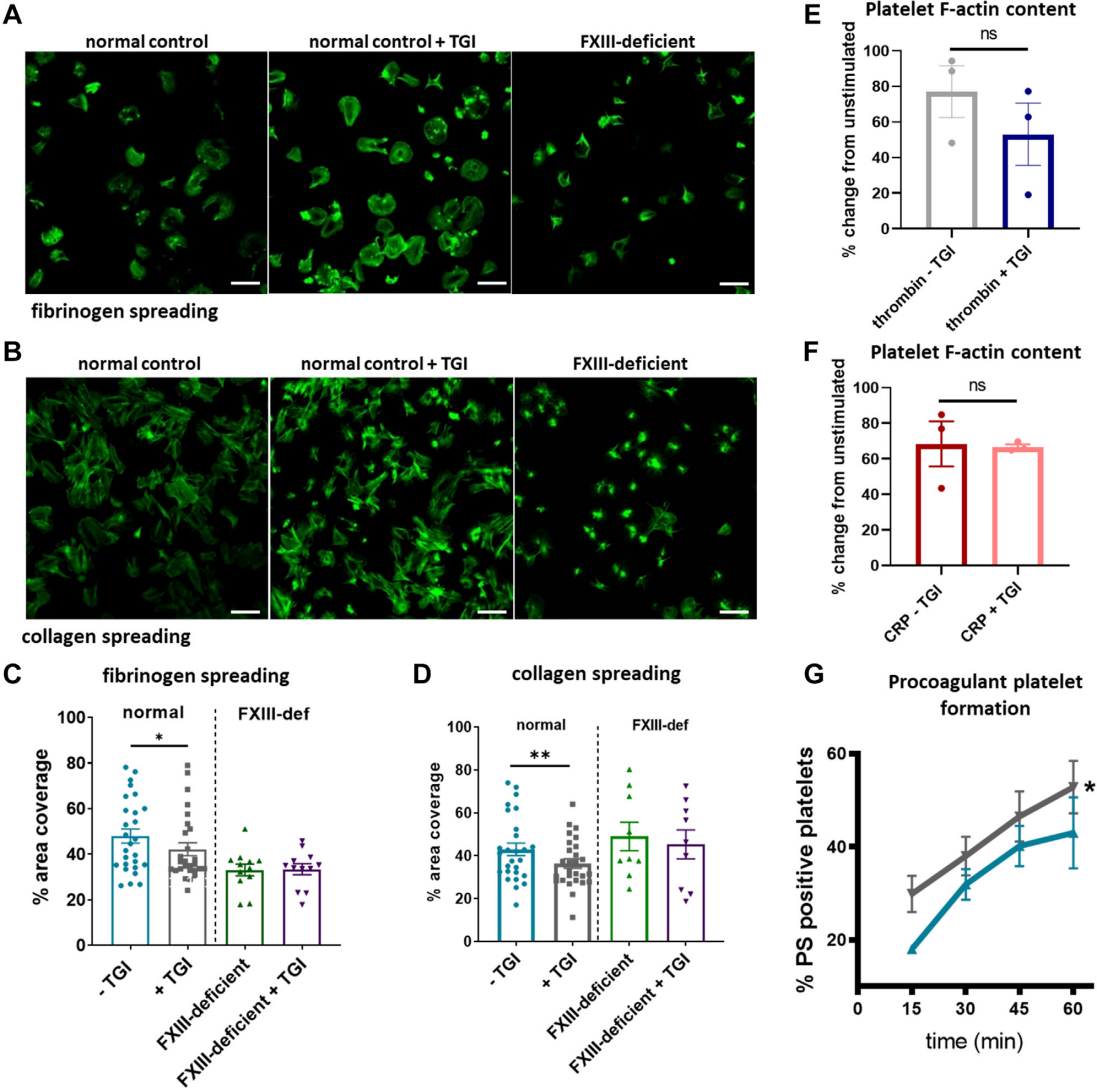
Inhibition of factor XIII-A reduces platelet spreading and limits procoagulant platelet formation, but does not affect actin polymerization. Normal platelets and factor XIII (FXIII)–deficient (FXIII-def) platelets were washed and incubated for 30 minutes in the presence or absence of transglutaminase inhibitor (TGI) before performing spreading studies at 37 °C for 60 minutes on either collagen (100 μg/mL) or fibrinogen (100 μg/mL). Platelets were stained using AF488-labeled phalloidin and imaged using fluorescence confocal microscopy. (A) Representative images of spread platelets on fibrinogen after 60 minutes from normal controls (left), normal controls + TGI (middle), and FXIII-deficient platelets (right). Scale bar represents 10 μm. (B) Representative images of spread platelets on collagen after 60 minutes from normal controls (left), normal controls + TGI (middle), and FXIII-deficient platelets (right). Scale bar represents 10 μm. (C) Platelet spreading on fibrinogen was measured by calculating the percentage area covered by platelets divided by the number of platelets in the field of view; 3 fields of view were taken per condition in each experiment and analyzed by a blinded investigator. Blue: normal platelets—TGI; gray: normal platelets + TGI; green: FXIII-deficient platelets; and purple: FXIII-deficient platelets + TGI. Statistical analysis was performed on normal platelet spreading where paired *t*-tests were used to determine significance between samples with and without TGI. ∗∗*P* < .01 and ∗*P* < .05. Normal donors (*n* = 10) and FXIII-deficient donors (*n* = 2; 2 separate experiment repeats per patient). (D) Platelet spreading on collagen was measured by calculating the percentage area covered by platelets divided by the number of platelets in the field of view; 3 fields of view were taken per experiment and analyzed by a blinded investigator. Statistical analysis was performed on normal platelet spreading where paired *t*-tests were used to determine significance between the sample with and without TGI. ∗∗*P* < .01. Normal donors (*n* = 10) and FXIII-deficient donors (*n* = 2). (E, F) Washed normal platelets were left resting or incubated for 30 minutes in the presence and absence of TGI before stimulating for 20 minutes with collagen-related peptide (CRP) (10 μg/mL; panel E) or thrombin (1 U/mL; panel F). Platelets were then fixed, permeabilized, and stained using AF488-labeled phalloidin to stain F-actin. F-actin was measured using flow cytometry where the median fluorescence intensity of AF488-phalloidin was used as a direct measurement of F-actin levels. Data were normalized as the percentage median fluorescence intensity of the unstimulated control and plotted as percent change from unstimulated control. Data show individual percent change values ± SEM. Statistical analysis was performed using Students *t*-test on percentage change data comparing samples −TGI to +TGI for each agonist. *n* = 3. (G) Normal platelets were washed and incubated for 30 minutes in the presence (gray) or absence (blue) of TGI before stimulating at 37 °C with a combination of cross-linked CRP (1 μg/mL) and thrombin (1 U/mL) for 15, 30, 45, and 60 minutes in the presence of 2 mM CaCl_2_. Phosphatidylserine (PS) was detected with Annexin V using flow cytometry and expressed using percentage positive ± SEM; statistical analysis was performed using paired *t*-tests on a separate area under the curve values. ∗*P* < .05. *n* = 4. ns, nonsignificant.

Given the close association of FXIII-A and actin and published observations that actin and other cytoskeletal proteins are FXIII-A substrates [[18], [19], [20]], we next addressed the impact of FXIII-A on platelet spreading on fibrinogen (Figure 2A, C) and collagen (Figure 2B, D). Inhibition of FXIII-A with TGI or the absence of FXIII-A in FXIII-deficient platelets impacted the extent of platelet spreading on fibrinogen, as measured by area coverage divided by the number of platelets per field of view. Inhibition or absence of FXIII-A had a more modest effect on collagen spreading where reductions in area coverage on collagen were observed in response to FXIII-A inhibition; however, no difference was observed in area coverage and platelet spreading in FXIII-A-deficient platelets. This suggests that the impact of FXIII-A on platelet spreading may be dependent on the adhesive surface and may be receptor-mediated or signaling pathway–mediated. Inhibition or absence of FXIII-A also reduced the ability of platelets to spread fully when adhered to fibrinogen or collagen, where many platelets had adhered but remained in a more compact confirmation with small protruding filopodia rather than the larger flatter appearance of fully spread platelets with lamellipodia (Figure 2C). To determine whether FXIII-A–meditated cytoskeletal effects during platelet activation occur via regulation of actin polymerization, flow cytometry was performed to measure F-actin content in the presence and absence of TGI following agonist stimulation. Despite our observations of reduced platelet spreading, no effect of FXIII-A inhibition on the formation of F-actin in stimulated platelets was detected (Figure 2E, F). These data indicate that platelet FXIII-A plays roles in the signaling pathways, leading to cytoskeletal rearrangement and shape change, but does not affect actin polymerization directly.

### 3.3 Platelet FXIII-A limits procoagulant platelet formation

We have previously described increased expression of (platelet) FXIII-A at the platelet surface in procoagulant platelets [17], and previous studies have implicated FXIII-A in the formation of procoagulant platelets [25,26]. To further elucidate the role of FXIII-A in platelet procoagulant activity, we measured procoagulant platelet formation in washed platelets from healthy volunteers in the presence and absence of TGI. Inhibition of platelet FXIII-A gave rise to a significant increase in the percentage of phosphatidylserine-positive platelets following dual stimulation with CRP-XL and thrombin (Figure 2G). This increase was consistent across all time points and suggests that platelet FXIII-A is involved in the suppression of procoagulant platelet formation.

### 3.4 FXIII-A supports platelet activation and its inhibition or absence attenuates fibrinogen binding to platelets

**Figure 3:**
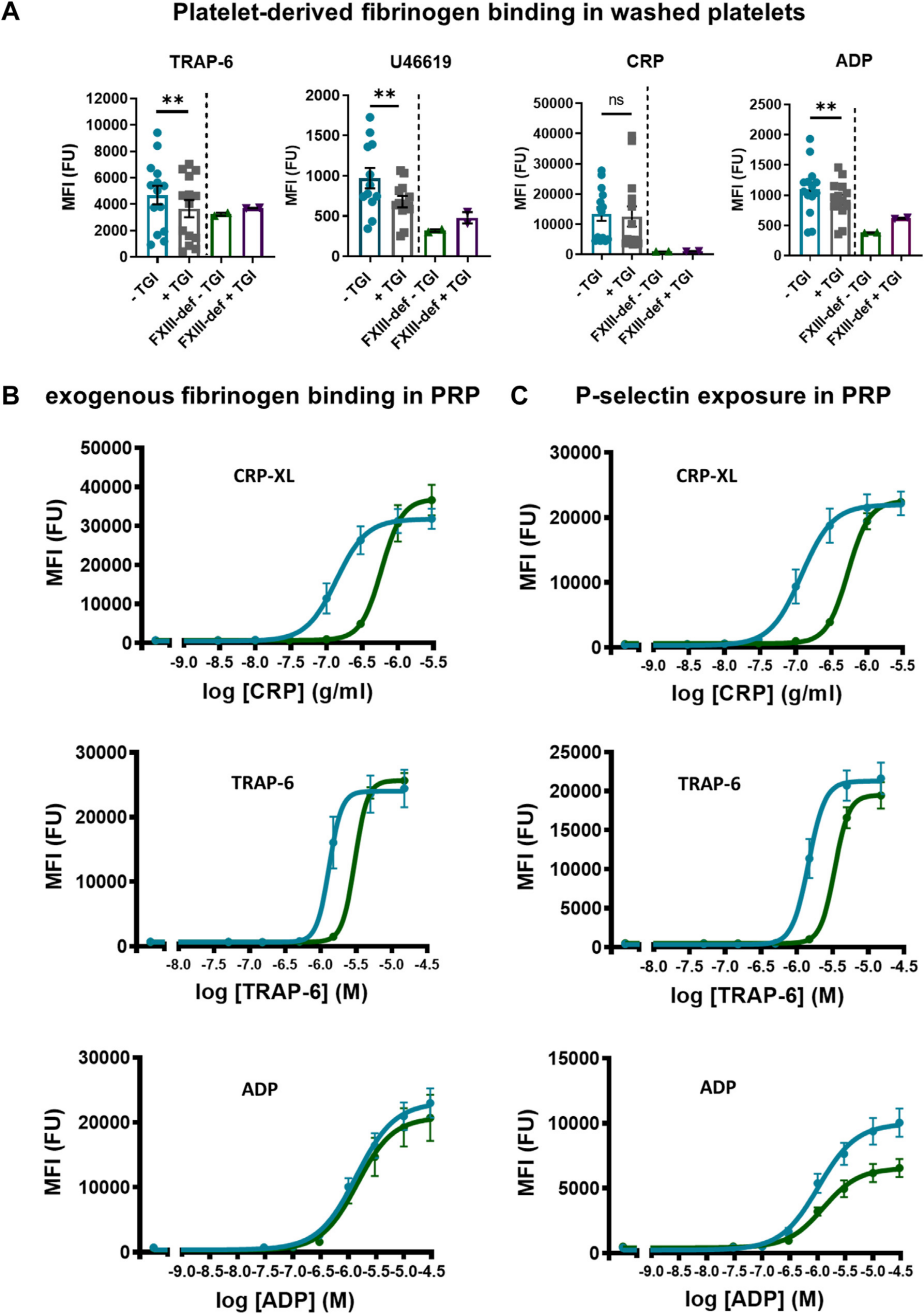
Platelet factor XIII-A promotes platelet activation and factor XIII-A inhibition or absence reduces platelet-derived fibrinogen binding to platelets. (A) Platelets were isolated and washed from normal healthy (blue) and factor XIII (FXIII)–deficient (FXIII-def) (green) individuals and incubated in the presence and absence of transglutaminase inhibitor (TGI) (normal: gray; (FXIII)-deficient: purple) for 30 minutes at 30 °C. Platelets were stimulated for 20 minutes at 37 °C with either thrombin receptor activator peptide 6 (TRAP-6) (5 μM; left), U46619 (1 μM; middle left), cross-linked collagen-related peptide (CRP-XL) (1 μg/mL; middle right), or adenosine diphosphate (ADP) (5 μM; right) in the presence of fluorescein isothiocyanate–labeled antifibrinogen antibody to detect fibrinogen binding. Data show individual median fluorescence intensity (MFI) values ± SEM of normal healthy controls (*n* = 14) and FXIII-deficient patients (*n* = 2). Statistical analysis in normal controls was performed and compared in samples with and without TGI using paired *t*-test, ∗∗*P* < .01. (B) Fluorescence intensity (FU) (MFI) for fibrinogen binding and (C) P-selectin exposure. Data represent mean FU ± SEM of responses to a range of concentrations of CRP-XL (0-3 μg/mL; top panels), TRAP-6 (0-15 μM) (middle panels), and ADP (0-30 μM) (bottom panels) in healthy controls (blue; *n* = 7) and FXIII-deficient patients (green; *n* = 2). CRP, collagen-related peptide; ns, nonsignificant; PRP, platelet-rich plasma.

Having observed a reduction in platelet outside-in mediated adhesion and spreading following inhibition or absence of platelet FXIII-A and considering evidence in the literature suggesting a relationship between extracellular FXIII-A and α_IIb_β_3_ [25,[30], [31], [32]], we next sought to determine whether platelet FXIII-A regulates integrin α_IIb_β_3_ activation and inside-out signaling. Fibrinogen binding to platelets in response to agonist stimulation is a measure of α_IIb_β_3_ activation and is largely variable within the normal population [28,33]. Pretreatment of washed platelets with TGI significantly reduced platelet-derived fibrinogen binding in response to TRAP-6, U46619, and ADP stimulation, but not CRP-XL, indicating both a reduction in α-granule secretion and integrin α_IIb_β_3_ activation. Similarly, platelets from FXIII-deficient patients showed marked attenuation of fibrinogen binding compared with that in normal platelets and no additional impact of TGI (Figure 3A).

Having identified a reduction in platelet-derived fibrinogen binding, we next assessed platelet activation in response to low-dose to high-dose agonist concentrations using flow cytometry to quantify the levels of both platelet- and plasma-derived fibrinogen binding to α_IIb_β_3_ (Figure 3B), a measure of α_IIb_β_3_ activation, and P-selectin exposure (Figure 3C), which is released upon platelet activation onto the platelet surface and is a measure of α-granule release. FXIII-deficient platelets in PRP from patients exhibited reduced sensitivity to agonist stimulation by CRP-XL and TRAP-6 (Figure 3B) compared with platelets in PRP from healthy donors. Interestingly, there was no reduction in sensitivity to ADP stimulation (Figure 3B). These data indicate that platelet FXIII-A is involved in the downstream signaling processes that convert agonist stimulation to α_IIb_β_3_ activation and P-selectin exposure via α-granule release.

### 3.5 Inhibition or absence of platelet FXIII-A reduces platelet thrombus formation on fibrinogen but not collagen

**Figure 4:**
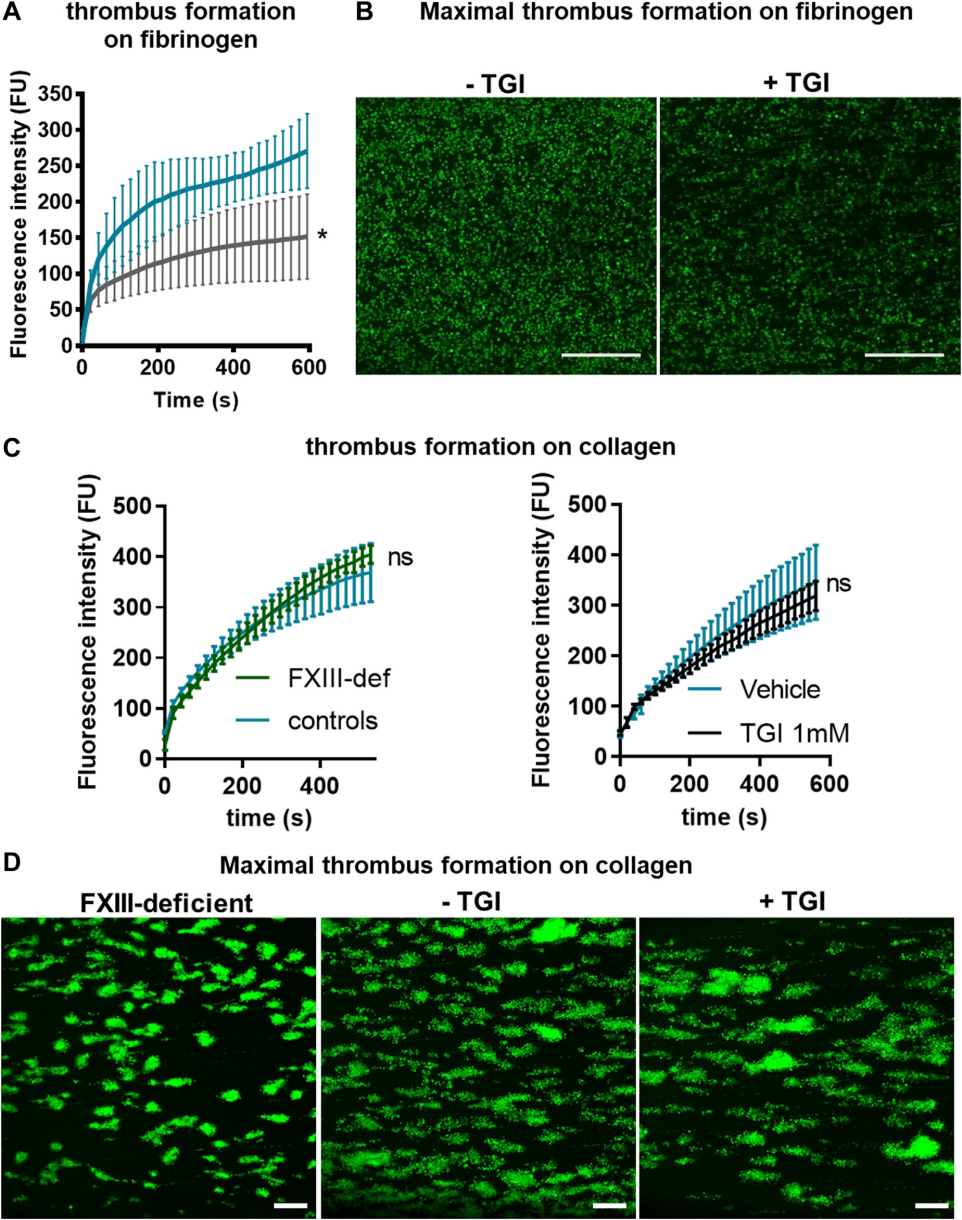
Platelet factor XIII-A inhibition or absence reduces platelet thrombus formation on fibrinogen but not collagen. (A, B) Whole-blood platelet thrombus formation under flow was performed with healthy donor blood at 500/s in capillary chambers coated with 100 μg/mL of fibrinogen with added DiOC6 (4 μg/mL) to label platelets and recorded over 600 seconds (s) using fluorescence confocal microscopy. (A) Data represents fluorescence intensity (FU) ± SEM of platelet deposition in the absence (blue) and presence (gray) of transglutaminase inhibitor (TGI) for over 600 seconds. Statistical analysis was performed on endpoint thrombus formation at the 600-second time point and compared in samples with and without TGI using paired *t*-test, ∗*P* < .05. *n* = 3. (B) Representative images of endpoint thrombus formation at the 600-second time point in healthy donor blood in the absence (left) and presence (right) of TGI. Scale bar represents 20 μm. (C) Platelet thrombus formation was performed at 1000/s on 100 μg/mL of collagen with whole blood from normal healthy donors (blue; left panel) and factor XIII (FXIII)–deficient (FXIII-def) donors (green; left panel); whole blood from normal healthy donors was also assayed in the absence (blue; right panel) and presence (black; right panel) of TGI for over 600 seconds. Data represent mean FU ± SEM. Statistical analysis was performed on endpoint thrombus formation at the 600-second time point and compared in samples with and without TGI using the paired *t*-test. Normal donors: *n* = 3; FXIII-deficient donors: *n* = 2. (D) Representative images of endpoint thrombus formation at the 600-second time point in FXIII-deficient patient blood (left) and healthy donor blood in the absence (middle) and presence (right) of TGI. Scale bar represents 20 μm. ns, nonsignificant.

Given the impact of FXIII-A on binding of fibrinogen to platelets, we next assessed whether it is involved in the adherence to and aggregation of platelets on immobilized fibrinogen under flow (500/s). Thrombus formation (median fluorescence intensity) on fibrinogen was significantly reduced upon pretreatment of normal platelets with TGI (Figure 4A, B). Interestingly, in contrast to fibrinogen, thrombus formation on collagen at 1000/s in blood from FXIII-A–deficient patients and healthy donors treated with and without TGI was similar, indicating that platelet FXIII-A does not contribute to platelets binding to and aggregating on collagen under shear rates of 1000/s (Figure 4C, D). These data suggest that the effects of FXIII-A on platelet thrombus formation may be receptor-specific and signaling pathway–specific.

### 3.6 Platelet FXIII-A enhances clot retraction

**Figure 5:**
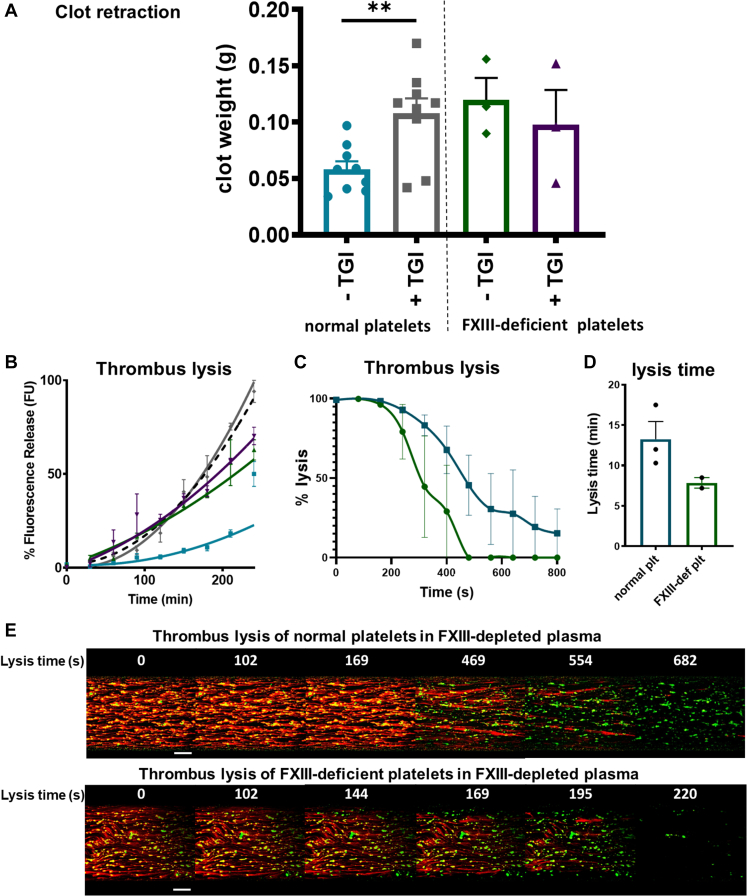
Inhibition or absence of platelet (plt) factor XIII-A reduces the extent of clot retraction and enhances the rate of fibrinolysis under flow. (A) Clot retraction was performed in nonsiliconized glass tubes using either normal or factor XIII (FXIII)–deficient (FXIII-def) washed platelets in FXIII-depleted plasma; clotting was initiated for 30 minutes with thrombin (1 U/mL) and CaCl_2_ (2 mM), and after 30 minutes, clots were imaged and weighed. Clot weight (g) was measured and compared in normal donors (*n* = 9) in the presence (gray) and absence (blue) of transglutaminase inhibior (TGI) and FXIII-deficient patients (*n* = 2 patients; 1 patient was recalled for a second time) in the presence (purple) and absence (green) of TGI. Data represents mean ± SEM of clot weight. Statistical analysis was performed on normal donors and samples with and without TGI compared using paired *t*-test; ∗∗*P* < .01. (B) Chandler model thrombi were formed from FXIII-deficient plasma (black dashed line) and the incorporation of either normal healthy washed platelets (blue) in the absence and presence (gray) of TGI or FXIII-deficient washed platelets (green) in the absence and presence (purple) of TGI. Data presented represents the median fluorescence intensity (FU) values normalized to the internal FXIII-deficient plasma control. FXIII-deficient patients (*n* = 2) and normal healthy controls (*n* = 2). (C) Thrombus formation under flow was performed in microcapillary chambers and visualized using fluorescence confocal microscopy. Whole reconstituted blood made up from FXIII-depleted plasma, either normal healthy platelets (blue) or FXIII-deficient platelets (green) with the red cells from the same donor in the presence of DiOC6 (4 μg/mL) to label platelets, AF647-labeled fibrinogen (75 μg/mL), and tPA (20 nM). Thrombi were fully formed at a shear rate of 500/second, which was achieved when fibrin and platelets covered the entire surface of the well before flow was switched to a lysis buffer containing 125 nM tPA in Tyrode’s buffer and thrombi were lysed to completion. Data represent the normalized FU values of AF647-fibrin(ogen) from the point at which full coverage was achieved and lysis was initiated to completion, where lysis times are represented in (D). Data represent normal healthy donors (*n* = 3) and FXIII-deficient patient donors (*n* = 2; mean ± SEM). (E) Representative images from various time points over the course of fibrinolysis: top images are from samples containing normal platelets in FXIII-depleted plasma and bottom images represent FXIII-deficient platelets in FXIII-depleted plasma. FXIII-deficient patient samples lysed faster, hence the shorter times on representative images. Fibrin(ogen): red; platelets: green. Scale bar represents 100 μm.

The aforementioned data demonstrate that FXIII-A partially colocalizes with actin and its inhibition or absence results in reductions in α_IIb_β_3_-mediated functional responses. We therefore investigated the contribution of platelet FXIII-A to clot retraction, a process that FXIII-A has previously been implicated in [[34], [35], [36]], which is also regulated by the platelet cytoskeleton, α_IIb_β_3_, and plasma FXIII [36] and is essential for clot stability. We performed assays with normal and FXIII-deficient platelets in FXIII-depleted plasma in the presence and absence of TGI. Clots formed from FXIII-deficient platelets or normal platelets in the presence of TGI retracted considerably less, as measured by an increased clot weight in the absence of platelet FXIII-A or upon its inhibition (Figure 5A). These data indicate a key role for FXIII-A in mediating clot retraction.

### 3.7 Platelets derived from FXIII-deficient patients are defective at stabilizing thrombi against fibrinolysis in the absence of plasma FXIII

During coagulation, plasma-derived FXIII [8,[37], [38], [39]] and platelet-derived FXIII-A [17,40] cross-link fibrin, stabilizing the fibrin clot and protecting it against fibrinolysis. We set out to confirm the role that platelet FXIII-A plays in this process using FXIII-A–deficient platelets in the absence of plasma FXIII-A. Compared to healthy controls, platelets from FXIII-deficient patients were unable to stabilize thrombi deficient in plasma FXIII against fibrinolysis (Figure 5B). To specifically assess the role of platelet FXIII-A in the stability of platelet-fibrin thrombi, whole blood was reconstituted with FXIII-A–deficient plasma and healthy donor platelets or FXIII-A–deficient platelets. Incorporation of FXIII-A–deficient platelets resulted in a faster rate of lysis than that seen in healthy donor platelets (Figure 5C–E). These data confirm that FXIII-deficient platelets lack the stabilizing effect of platelets from normal individuals in FXIII-depleted thrombi.

## 4 Discussion

This study has built upon our previous work [17] to confirm that platelet FXIII-A bridges the gap between platelets and coagulation, having simultaneous roles in the intracellular and extracellular clot environment. The current study shows that cellular FXIII-A internally drives platelet activation and clot retraction, in addition to its extracellular roles of stabilizing thrombi against fibrinolysis [17], which we demonstrated previously. Here, we have shown that platelet FXIII-A has intracellular roles in platelet function, including platelet spreading, integrin α_IIb_β_3_ and fibrinogen binding, and α-granule secretion. We have demonstrated the crucial role of platelet FXIII-A in platelet activation, where FXIII-A influences the sensitivity of platelets to agonist stimulation and regulates the phenotypic change that platelets undergo to become procoagulant. We show herein that FXIII-A mediates binding of fibrinogen to activated platelets. Furthermore, FXIII-A is active within stimulated platelets and its activity is well-dispersed throughout the whole platelet. We have established that FXIII-A is involved in cytoskeleton-mediated processes, where it plays roles in the regulation of platelet spreading and in clot retraction.

The data presented herein show that upon platelet activation, FXIII-A is present and active within the platelet cytoplasm, indicating that the platelet pool of FXIII-A is only partially exposed on the stimulated platelet surface, as suggested in our previous work [17], and is partially retained intracellularly to perform roles in platelet activation. Here, we demonstrate the importance of intracellular FXIII-A in the binding of platelet-released and extracellular fibrinogen to platelets and evidence the link between the intracellular and extracellular mechanisms of FXIII-A. Platelet binding to fibrinogen under static and flow conditions is reduced significantly upon the inhibition or absence of platelet FXIII-A. It is worth noting that the data in Figure 3A measure the binding of platelet-released fibrinogen to the platelet surface and the experiments in Figure 3B measure plasma- and platelet-derived fibrinogen binding to the platelet surface. Interestingly the differences between healthy donor and patient fibrinogen binding are less pronounced at higher agonist concentrations in the presence of plasma FXIII in Figure 3B. This suggests that platelet FXIII-A mediates the binding of α-granule fibrinogen to the platelet surface and that its role in plasma fibrinogen binding may be via its influence on platelet activation and receptor externalization, which is more apparent at lower agonist concentrations. The receptor density of α_IIb_β_3_ was neither measured in this study nor compared between patients and normal healthy controls; thus, another possible explanation of reduced fibrinogen binding to FXIII-deficient platelets could be a reduced surface expression or externalization of α_IIb_β_3_, which should be addressed in future studies. Our study demonstrates a strong relationship between FXIII-A and α_IIb_β_3_; these observations are supported by previous studies demonstrating that plasma FXIII and platelet FXIII-A are associated with α_IIb_β_3_. Previous studies have demonstrated that exogenous FXIII can bind to the platelet surface via α_IIb_β_3_ [41], which facilitates adhesion to FXIII coated surfaces under static and flow conditions [31] potentially via receptor-bound fibrin(ogen) [30]. Endogenous platelet FXIII-A, alongside calpain, regulates the adhesive function of α_IIb_β_3_ in response to sustained intracellular calcium [25], which supports our observations of reduced platelet responses following adhesion to fibrinogen. FXIII-A also acts synergistically with α_IIb_β_3_ to initiate platelet-associated fibrin formation [32] and support clot retraction, and it cross-links extracellular α_2_-antiplasmin to fibrin to stabilize thrombi against fibrinolysis [17]. Together with our study, these data indicate that platelet FXIII-A is involved in α_IIb_β_3_ activation and α_IIb_β_3_-mediated platelet function responses.

FXIII-A plays a key role in the control of platelet signaling. Our data demonstrate that FXIII-A enhances platelet signaling, where platelets containing no FXIII-A exhibit decreased sensitivity to agonists, thus requiring higher concentrations to activate to the same extent as normal platelets. The measurement of platelet sensitivity is a novel concept proposed in our recent study [28]. Here, we show that the sensitivity of the FXIII-deficient platelets assessed herein is considerably reduced when compared to normal platelets, suggesting that FXIII-A is involved in downstream signaling processes occurring between receptor sensitization, degranulation, and α_IIb_β_3_ activation.

The impact of both platelet and plasma FXIII-A on clot retraction has been previously studied where results have been conflicting, likely due to experimental timing differences and the contribution of other exogenous factors such as plasma FXIII and red blood cells [36,42]. We performed clot retraction in a FXIII-free system in the absence of red blood cells to tease out the exclusive role of platelet FXIII-A in this process and showed that clot retraction was reduced upon inhibition or absence of platelet FXIII-A. Similarly, Kasahara et al. [34,35] demonstrated that clot retraction was reduced in PRP from FXIII-deficient mice and its effects were mediated via lipid rafts and α_IIb_β_3_ linking the extracellular fibrin network to the intracellular actin cytoskeleton. Plasma FXIII is important in regulating the clot size and density via its extracellular cross-linking of fibrin α-chains, which enhances red cell retention [42]. The contribution of platelet FXIII-A to clot retraction is contested by Kattula et al. [36], who reported that platelet FXIII-A has minimal impact; however, this study used a longer time point than that used in the present study, and it is possible that the effects of platelet FXIII-A were overlooked. This suggests that perhaps platelet FXIII-A plays a greater role in the initial stages of clot compaction, where its influence on actin dynamics and fibrinogen binding to α_IIb_β_3_ are important contributors.

We show that actin partially colocalizes with FXIII-A antigen and activity in stimulated platelets and that FXIII-A inhibition or absence reduced platelet spreading, another process mediated by the actin cytoskeleton. FXIII-A inhibition, however, has no direct effect on actin polymerization, suggesting its influence on actin dynamics is via signaling pathways that regulate platelet shape change. FXIII-A contributes to osteoclast shape change by regulating RhoA [43], a molecule also involved in cytoskeletal rearrangement in platelets [[44], [45], [46]]. Therefore, it is plausible that FXIII-A–mediated cytoskeletal effects may occur via RhoA; however, future studies exploring this are required. We show that FXIII-A is present in actin nodules, which form during the early stages of platelet adhesion and spreading [47], play roles in clot retraction and thrombus formation, and are involved in platelet aggregate formation under flow [48]. The partial colocalization of FXIII-A with the actin cytoskeleton suggests that it is also possible that platelet FXIII-A is involved in stabilization of these structures to influence platelet shape change, thrombus structure, and clot retraction, or alternatively that actin may regulate the localization and activity of FXIII-A. Our data also demonstrate that the inhibition or absence of FXIII-A has different effects on the binding and subsequent activation and aggregation of platelets on collagen under flow when compared to static platelet adhesion and spreading. We observed no effect in platelet thrombus formation assays in the absence of coagulation, suggesting that platelet FXIII-A is not involved in platelet adherence to collagen, activation, and aggregation under shear stress but does play a more important role in stabilizing thrombi in the presence of coagulation, as demonstrated in fibrinolysis assays. Platelet spreading on collagen under static conditions, however, was modestly reduced upon FXIII-A inhibition but not in its absence, but similar to that observed under flow, adhesion to collagen was not affected. Together, these data suggest that FXIII-A may be involved in platelet shape change via intracellular signaling and actin-mediated processes during platelet spreading but not in the receptor-mediated binding of platelets to collagen via the GPVI receptor under both static and flow conditions.

We recognize that a limitation of this work is the small number of FXIII-A patients included in this study. The number of FXIII-deficient patients in the UK is low and access to such patients is rare; therefore, obtaining large numbers of patients for a study is simply not feasible. This highlights the importance of using patient blood samples to maximum effect to further our understanding of FXIII-A, as was the objective in this study. Although greater patient numbers would strengthen the conclusions, the FXIII-A–deficient platelet data obtained in this study show a clear reduction in multiple aspects of platelet function in the absence of FXIII-A, which can be used to inform future investigations in the role of platelet FXIII-A.

In conclusion, we have demonstrated a clear role for intracellular platelet FXIII-A in mediating platelet activation and clot retraction. We have shown that FXIII-A is integral in regulating the sensitivity of platelets to agonist stimulation, procoagulant platelet formation, and the binding of fibrinogen to α_IIb_β_3_. These data confirm that platelet FXIII-A is an important regulator of platelet function and highlight its relevance as an effective therapeutic target in thrombosis.
