# Supplementary material for: Platelet factor XIII-A regulates platelet function and promotes clot retraction and stability
Source: Res Pract Thromb Haemost. 2023 Jun 7;7(5):100200. doi: 10.1016/j.rpth.2023.100200 (PMC10439398; doi:10.1016/j.rpth.2023.100200)
Supplement: Supplemental Information and Figures [file mmc1.docx]

**Supplemental information and figures**

**Mass spectrometry sample preparation method**

Mass spectrometry was performed on isolated platelet cytoplasmic contents from resting platelets that had been treated with either 100 mM TGI for 30 mins or with a vehicle control (dH_2_O). Platelets were washed as described in the methods section prior to a 30 min treatment with 1 mM TGI. Platelets were washed extensively to ensure removal of all TGI from the buffer and platelet surface by diluting 1:15 with modified Tyrode’s-HEPES buffer prior to a further 2 x 10 min centrifugation steps at 1413 x g in the presence of 1.25 μg prostaglandin I2 and 1:3 v/v ACD. Platelet cytoplasm suspensions were counted and adjusted to 4x10^8^/ml before platelet lysis was initiated by freeze thawing (-80^o^c to 37^o^c) 3 times before ultracentrifugation at 100,000g (55000rpm) using a Beckman Coulter ultracentrifuge with rotor SW55). Platelet cytoplasm was collected as the supernatant and filtered through a 0.22 μM filter before mass spectrometry analysis.

**Mass spectrometry method**

5 µL of sample or standard was injected onto a Waters reversed-phase column (ACQUITY UPLC BEH Shield RP18 Column, 130Å, 1.7 µm, 2.1 mm X 150 mm) via a Thermo Accela UPLC. The autosampler was kept at 20 °C and column oven at 60 °C. Mobile phase A was water with 0.1% formic acid and mobile phase B was acetonitrile with 0.1% formic acid. The gradient, as %B, was conducted at 200 µL/min and was as follows: 0 mins = 5%, 2 mins = 5%, 8 mins = 10%, 9 mins = 80%, 12 mins = 80%, 12.5 mins = 5%, 20 mins = 5%. The flow was directed into a Thermo Scientific LTQ-Orbitrap XL operating in full-scan positive ion mode. The resolution was set at 15,000, the mass range was 85-500 m/z and n-butyl benenesulfonamide plasticizer (214.089626) was set as an internal lock mass.

**Mass spectrometry results**

The TGI standard comes out at 3.1 min, in the cytoplasm of platelet treated with TGI it comes out slightly later than this at 4.0 min, most likely due to the presence of chromatographically retained undesirable debris (crud). There is no TGI detected in the samples where platelets were treated with vehicle control. These data show that TGI can be detected in the cytoplasmic contents of platelets that have been incubated with TGI and subsequently washed, indicating that TGI crosses the platelet membrane to gain access to the platelet cytoplasm.

**
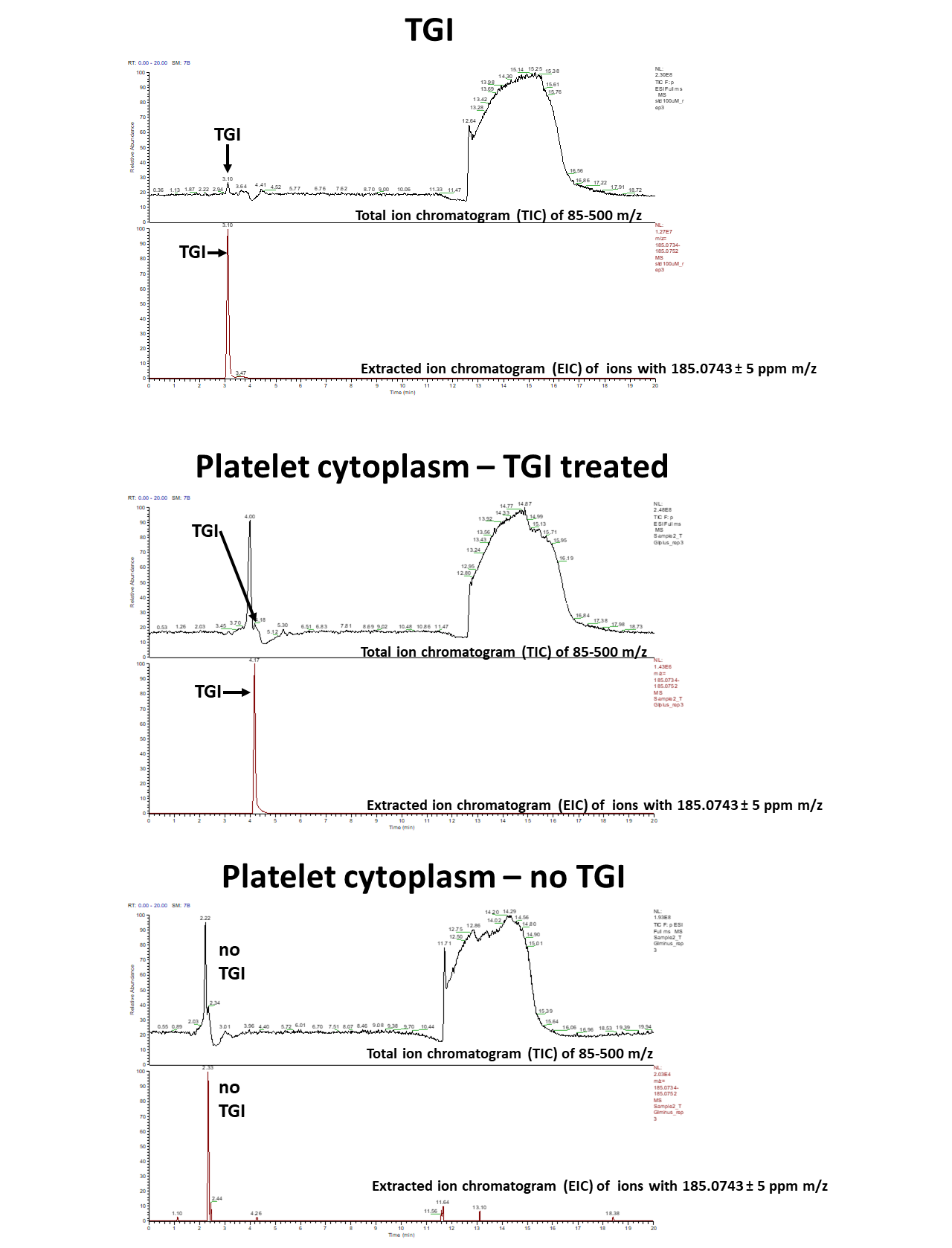
**

**Supplemental Figure.** Mass spectrometry was performed on 100 mM TGI and compared with isolated cytoplasm from platelets that had previously been incubated with 100 mM TGI or vehicle control. Figure shows the output of TGI run alone (top), cytoplasm from platelets incubated with TGI (middle) and cytoplasm of platelets incubated with vehicle (bottom). Data is representative plots from 3 normal healthy donors.
